# Studying Biofilm and Clinical Issues in Orthopedics

**DOI:** 10.3389/fmicb.2019.00359

**Published:** 2019-02-26

**Authors:** Trisha N. Peel

**Affiliations:** ^1^Department of Infectious Diseases, Monash University, Melbourne, VIC, Australia; ^2^Alfred Health, Melbourne, VIC, Australia

**Keywords:** biofilm, prosthetic joint infection, anti-biofilm approaches, diagnosis, treatment, prevention

## Abstract

The association between biofilm-forming microorganisms and prosthetic joint infection influences all aspect of management including approaches to diagnosis, management and prevention. This article will provide an overview of new anti-biofilm strategies for management of prosthetic joint infection.

## Introduction

Implantation of prosthetic joints exemplifies the clinical challenges for the management and prevention of biofilm associated medical device infections. Biofilm forming microorganisms include bacteria, such as *Staphylococcus aureus* and *Staphylococcus epidermidis*, and some fungal species, such as *Candida albicans* (Benito et al., 2016). Prosthetic joint replacement surgery is a common surgical procedure with over one million procedures performed each year in the United States (Etkin and Springer, 2017). The incidence of prosthetic hip and knee infection is 1–3% and infection is a major indication for revision arthroplasty (Kurtz et al., 2007). Modeling data predicts the incidence of prosthetic joint infection will increase to greater than 6% by 2030 (Kurtz et al., 2007), owing to factors such as increased demand for surgery, the aging population and the obesity epidemic (Kurtz et al., 2007; Adams et al., 2008; George et al., 2017).

Given the central role biofilm plays in prosthetic joint infections, approaches that target the biofilm are critical for the successful diagnosis, management and prevention of prosthetic joint infections.

## Diagnosis

The detection of prosthetic joint infection requires a high degree of clinical acumen as the protean symptoms of infections, such as fever, are frequently absent (Inman et al., 1984; Barrett and Atkins, 2014). Conventional microbiological culture techniques frequently fail to detect biofilm associated microorganisms (Atkins et al., 1998). Novel strategies have been developed to increase the yield of detection of biofilm-embedded microorganisms. Sonication is one such strategy that dislodges microorganisms from the biofilm and prosthesis surface through the application of low-frequency ultrasonication to the resected prosthetic device (Trampuz et al., 2007; Tande and Patel, 2014; Koo et al., 2017). A number of trials have demonstrated improved sensitivity for the diagnosis of infection with sonication, compared with standard microbiological culture techniques (Trampuz et al., 2007; Piper et al., 2009; Zhai et al., 2014). An intermediate vortexing step is frequently applied which creates microbubbles to increase interfacial tension promoting further dislodgement of biofilm (Trampuz et al., 2007; Piper et al., 2009; Zhai et al., 2014; Koo et al., 2017).

There also has been interest in the use of chemical agents to disperse biofilm. Dithiothreitol (DTT) is a reducing agent that has been compared to sonication with similar sensitivity between the two techniques (Sambri et al., 2018). Sonication and chemical techniques for biofilm dislodgement are cost-effective compared to conventional culture techniques, particularly when the impact of missed diagnoses are taken into account (Romano et al., 2018).

Both techniques, however, are performed on a resected prosthesis and may not be available if debridement and implant retention approaches are undertaken (Peel et al., 2016). Other research has focussed on strategies to optimize microbiological culture techniques through the inoculation and culture of periprosthetic tissue specimens in blood culture bottles (Hughes et al., 2011; Peel et al., 2016) The diagnostic accuracy of periprosthetic tissue culture in blood culture bottles appears to be comparable with sonication fluid culture (Yan et al., 2018).

Despite these advances, the diagnosis may still be missed in up to 30% of cases of prosthetic joint infection (Hughes et al., 2011; Zhai et al., 2014; Peel et al., 2016; Romano et al., 2018). Non-culture techniques such as DNA sequencing and next generation sequencing have been investigated to improve the rate of organism detection (Portillo et al., 2012; Cazanave et al., 2013; Tarabichi et al., 2018). In addition, there has been increasing interest in biomarkers to augment the diagnosis of infections. The most promising is alpha-defensin, an antimicrobial peptide produced by the innate immune system (Ganz, 2003). The test was originally developed as an enzyme-linked immunosorbent assay (ELISA) then later developed into a lateral flow test kit as a point-of-care test (Marson et al., 2018). Early studies reported very high sensitivities of 97–100%, including for bacterial and fungal prosthetic joint infections (Deirmengian et al., 2015; Wyatt et al., 2016) however, more recent studies have reported lower sensitivity. Other research has suggested the sensitivity of alpha defensin may not differ from established diagnostic techniques including histopathology, microbiological culture or serum C-reactive protein (Sigmund et al., 2017). Furthermore, the use of the lateral flow kits may have lower accuracy compared with the ELISA techniques (Suen et al., 2018). The results of a large, definitive clinical validation study (ClinicalTrials.gov Identifier: NCT02868736) comparing the ELISA and lateral flow test in 3000 patients has recently completed recruitment and the results will provide important data on the test performance.

## Treatment

The goals of prosthetic joint treatment are 2-fold: to remove the biofilm-associated pathogens whilst maintaining a functional, pain-free joint (Zimmerli et al., 2004). As with diagnosis, treatment options are influenced by the presence of biofilm. It is well recognized that, in chronic prosthetic joint infections, where there is an established, mature biofilm, removal of the prosthesis is currently the only approach to cure the prosthetic joint infection (Zimmerli et al., 2004; Koo et al., 2017). A new prosthesis may be re-implanted as part of a one- or two-stage exchange (Zimmerli et al., 2004). For acute infections, where the biofilm is immature, it may be possible to cure the infection without removal of the prosthesis; so called “debridement and retention” strategies (Zimmerli et al., 2004). Debridement and retention is an attractive option, in appropriately selected patients, as it is associated with better patient outcomes and reduced costs (Peel et al., 2013b; Aboltins et al., 2016). This strategies relies on the physical removal of the biofilm through surgical debridement and lavage of the prosthesis, combined with antimicrobials.

The selection of antimicrobials for debridement and retention is critical. Treatment of biofilm demonstrates mechanisms of resistance and/or tolerance to many antimicrobials (Lebeaux et al., 2014). Mechanisms for this “recalcitrance” (Lebeaux et al., 2014) include impaired penetration of antimicrobials through the extracellular matrix (Campanac et al., 2002), reduced metabolic activity and cellular turnover of biofilm-embedded microorganisms (Sternberg et al., 1999) and, presence of subpopulations of slow-growing or quiescent microorganisms termed “persister cells” and/or “small-colony variants” (Conlon et al., 2015; Waters et al., 2016; Koo et al., 2017). Antimicrobials such as rifampicin, have higher activity against biofilm-associated organisms, particularly *Staphylococcus* species and *Streptococcus* species both in *in vitro* and clinically (John et al., 2009; Peel et al., 2013a; Lora-Tamayo et al., 2017). The main limitation with Rifampicin, is the low barrier to the emergence of resistance when used as a single agent (Koo et al., 2017). Over recent years, there has been increasing interest in antimicrobial peptides; the term refers to a group of small molecules with activity against a broad range of microorganisms (Koo et al., 2017). Many of these agents are in pre-clinical testing, however, one antimicrobial peptide, colistin, is currently used in clinical practice, particularly for the management of multi-resistant Gram-negative organisms (Corvec et al., 2013; Jochumsen et al., 2016). Colistin has demonstrated anti-biofilm activities, particularly when combined with other antimicrobials such as fosfomycin (Corvec et al., 2013). The poor toxicity profile and risk of emergence of resistance has limited the use of colistin, however, it remains a treatment option for Gram-negative biofilm infections (Corvec et al., 2013). In fungal prosthetic joint infections, echinocandins, and amphotericin appear to have better biofilm activity when compared to azoles (Kuhn et al., 2002). However, both debridement and retention and exchange strategies have poorer outcomes with fungal prosthetic joint infection compared with bacterial infections and many guidelines recommend resection of the prosthesis in the setting of fungal infections (Azzam et al., 2009; Pappas et al., 2016).

A number of strategies have been examined *in vivo* to augment the activity of antimicrobials such as the use of agents to disrupt or disperse the extracellular matrix, and bacteriophage therapy, however, these strategies have not been translated into clinical practice for the management of prosthetic joint infections (reviewed in Lebeaux et al., 2014; Hogan et al., 2015; Koo et al., 2017).

## Prevention

Given the adverse consequences and costs, the adage “*prevention is better than cure”* is pertinent for prosthetic joint surgery. Prevention strategies include standard infection control practices relevant for all surgical procedures such as surgical site skin preparation with an alcohol-based antiseptic, surgical hand antisepsis, screening and decolonisation for *S. aureus* and, appropriate and timely surgical antimicrobial prophylaxis (Allegranzi et al., 2016a). The World Health Organization and the Centers for Disease Control and Prevention (CDC) have recently published guidelines for the prevention of surgical site infections which provide a comprehensive overview of the evidence for these prevention strategies (Allegranzi et al., 2016a,b; Berrios-Torres et al., 2017) In addition, the CDC guidelines included a separate section examining the evidence for seven key questions for infection prevention following joint replacement surgery, including a question focussed on effective strategies to prevent biofilm formation (Berrios-Torres et al., 2017). The authors of the CDC guidelines examined the impact of cement or prosthesis modification, vaccination and novel biofilm control agents, however, were unable to provide recommendations given the limited evidence available in these areas (Berrios-Torres et al., 2017).

Antibiotic-loaded cement is frequently used for fixation of the prosthesis and to facilitate elution of antimicrobials to prevent early biofilm formation. Four randomized controlled trials have been conducted to examine the impact of antibiotic-loaded cement in prosthetic joint surgery (McQueen et al., 1990; Josefsson and Kolmert, 1993; Chiu et al., 2001, 2002). Two studies compared systemic antibiotics to antibiotic loaded cement and did not demonstrate a difference in infection risk (McQueen et al., 1990; Josefsson and Kolmert, 1993). Two studies (performed by the same group), examined the addition of antibiotic-loaded cement to systemic antibiotic prophylaxis and demonstrated a reduction in the incidence of prosthetic joint infection with the addition of antibiotic-loaded cement (Chiu et al., 2001, 2002). These studies had a number of methodological issues with risk of bias. The meta-analysis performed by the CDC guidelines group, concluded that there were “*uncertain trade-offs between the benefits and harms*” and no recommendation was provided (Berrios-Torres et al., 2017). Importantly, the most common antibiotics incorporated into cement are aminoglycoside antibiotics, whereas only one trial has examined gentamicin (Josefsson and Kolmert, 1993), all other randomized controlled trials examined the incorporation of cefuroxime into cement (Birkeland et al., 2017). Despite this, the use of antibiotic-loaded cement is routinely performed in many centers (International Consensus Meeting on Periprosthetic Joint Infection, 2013). Data from large, national registries have demonstrated reduced risk of infection with the use of antibiotic-loaded cement.(Engesaeter et al., 2006; Bohm et al., 2014) Of note, however, the registries also demonstrated an increased rate of aminoglycoside resistance, which may be attributed to the use of aminoglycoside antibiotic-loaded cement (Lutro et al., 2014).

Modification of the prosthesis surface is another strategy to prevent biofilm formation, including coating with antimicrobials, silver or other metals (Alt, 2017). Silver coated endoprostheses have been investigated in small retrospective studies in patients undergoing surgery for bone and soft tissue cohort (Alt, 2017). This cohort of patients have a markedly increased risk of prosthesis infection (Peel et al., 2014; Wafa et al., 2015; Hardes et al., 2017). The use of silver-coated prosthesis is associated with reduction in infection in these small, uncontrolled studies (Alt, 2017). The occurrence of local argyria, a blue discoloration of the skin due to accumulation of silver in local tissues has been reported (Alt, 2017).

The final prevention strategy that has been extensively investigated is vaccination strategies targeting *S. aureus*. Despite over five-decades of research, vaccines targeting *S. aureus* have not been successfully translated into clinical practice (Giersing et al., 2016). The challenges impeding the progress of vaccine development have been well described (Giersing et al., 2016; Mohamed et al., 2017). The STRIVE Trial (ClinicalTrials.gov:NCT02388165), a Phase 2b trial, is comparing the safety and efficacy of the SA4Ag Vaccine (Pfizer) for the prevention of *S. aureus* blood stream infections and/or deep surgical site infections in participants undergoing spinal implantation surgery. This trial aims to recruit 6000 participants with the planned completion data of August 2019. If successful, this trial may have implications for preventative strategies for other surgical site infections, including prosthetic joint surgery.

## Conclusion

The inclusion of biofilm-focused strategies for arthroplasty surgery in the Centers for Disease Control and Prevention Surgical Site Infection guidelines highlight the prominence of this clinical entity. Understanding the link between biofilm and arthroplasty infections has driven research into novel strategies to diagnose, treat and prevent these infections, with the promise of significant advancements in this field in the coming years.

## Author Contributions

TP was involved in all aspects of the manuscript.

## Conflict of Interest Statement

The author declares that the research was conducted in the absence of any commercial or financial relationships that could be construed as a potential conflict of interest.
